# High-throughput synthesis of nanoparticles using oscillating feedback microreactors: a selective scaling-out strategy†

**DOI:** 10.1039/d3ra08037d

**Published:** 2023-12-11

**Authors:** Mingxin Li, Wensheng Wang, Cong Xu

**Affiliations:** a Tsinghua University, Institute of Nuclear and New Energy Technology Haidian District Beijing China c-xu@mail.tsinghua.edu.cn

## Abstract

High-throughput synthesis of high-quality nanoparticles using traditional methods is difficult because of the complexities in controlling residence time and concentration. Passive oscillating feedback micromixers (OFMs) can overcome these difficulties. However, it remains a challenge to significantly increase throughput while retaining the microfluidic advantages. In this work, a selective scaling-out strategy was used to generate a series of enlarged OFMs, which were demonstrated to produce BaSO_4_ nanoparticles. A novel chaotic convection synthesis mode was found to produce high-quality BaSO_4_ nanoparticles (mean size, 24.91 nm; size distribution, 10–50 nm) at a high throughput of 281.4 mL min^−1^. The NP production rate can reach 538.4 g h^−1^, far exceeding the reported production rates.

## Abstract 2

High-throughput synthesis of high-quality nanoparticles using passive and chaotic oscillating feedback microreactors to be selectively scaled out.

## Graphical abstract

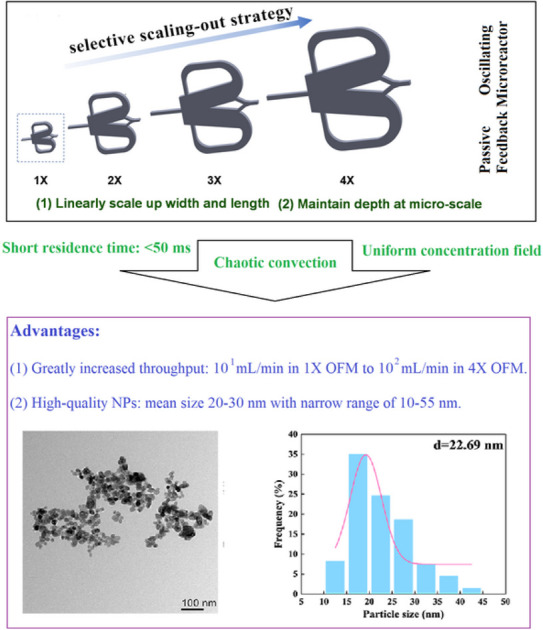

## Introduction

In general, NPs with small mean size and narrow size distribution are preferred because of their excellent physicochemical properties.^1^ NPs can be synthesized in a liquid system through two stages: nucleation and crystal growth.^2,3^ In a traditional batch reactor, the two stages occur in the same area, making it difficult to precisely control the time of each stage. This problem results in aggregation and wide-size distribution of the synthesized NPs. Microreactors are considered to solve this critical problem because they can precisely control the residence time and concentration field of the NP synthesis. These ordered-flow microreactors can be divided into two categories: continuous-flow microreactors and segment-flow microreactors. Continuous-flow microreactors can improve the uniformity of reaction and operate continuously,^4,5^ while segmented-flow microreactors can enhance mixing and eliminate axial dispersion.^6,7^ Steady, ordered, and controllable flows are the distinctive features of these two types of microreactors. However, to maintain the delicate flow patterns stably, the reactant throughput must be low enough to avoid a strong shear force. Thus, the above-mentioned microreactors reported for NP synthesis should be worked at low throughputs of 0.2 mL min^−1^–10 mL min^−1^ (Table S1†).

Recently, a passive micromixer, *i.e.*, an oscillating feedback micromixer (OFM) has attracted much attention because it can be operated at Reynolds numbers (Re) of up to a magnitude of 10.^2,8,9^ OFMs which are based on the Coanda effect can generate a high-frequency oscillation and induce intense chaotic convection thereby enabling rapid mixing and mass transfer.^10^ At present, the maximum throughput of OFMs used for NP synthesis can reach 160 mL min^−1^, which is much higher than that of continuous flow microreactors and segment flow microreactors.^11^ However, the large-scale commercial production of NPs requires a much higher throughput with a production rate of at least 100 g-NPs/h. In practice, a significant throughput increase for one microreactor is not feasible due to the significant increase in flow resistance. Thus, the numbering-up strategy has been used for microreactors to increase throughput.^12,13^ This strategy requires a complex tree-like or ladder-like network to distribute feeds into multiple identical microreactors. The parallel design can increase the throughput linearly to a certain level.^14,15^ However, increasing the number of complicated microchannels significantly increases machining and working costs.^16^ And it is very difficult to keep the fluids flowing uniformly through each microreactor all the time. Thus, the numbering-up strategy is inappropriate for the OFM scale-up. Until now, it has been a challenge to synthesize high-quality NPs using microreactors while maintaining high throughput productivity.

In this paper, a selective scaling-out strategy, which is different from the numbing-up strategy, was presented to achieve a significant increase in the throughput of OFMs. The scaled-out OFMs were used to demonstrate the synthesis of BaSO_4_ NPs. The flow patterns, synthesis dynamics, and NP properties were experimentally investigated. The results proved that the selective scaling-out strategy is valid for the high-throughput and high-productivity synthesis of NPs with small and narrow size distribution.

## Experimental

### Oscillating feedback micromixer

**Fig. 1:**
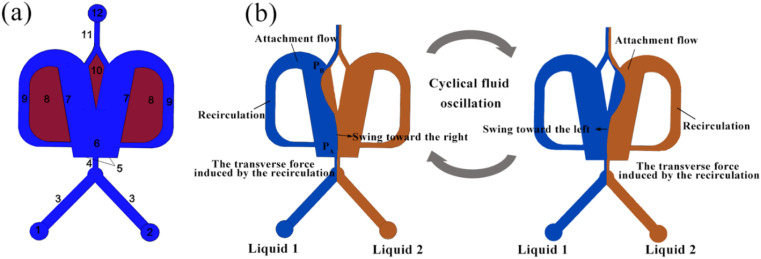
Oscillating feedback micromixer: (a) structure (1 and 2: inlet port; 3: Y-type feed channel; 4: confluence channel; 5: Coanda step; 6: mixing chamber; 7: attachment wall; 8: barrier; 9: feedback flow channel; 10: splitter; 11: outlet channel; 12: outlet port) and (b) efficient micromixing mechanism.

Fig. 1(a) illustrates the schematic of an OFM, which mainly consists of an inlet channel (1–4), a mixing chamber (6), two feedback flow channels (9), a splitter (10), and an outlet channel (11 and 12). Fig. 1(b) illustrates the working mechanism of the OFM using the Coanda effect, *i.e.*, the attachment flow phenomena.^15^ When two different fluids enter the suddenly expanded mixing chamber from a narrow inlet channel, a jet flow is formed, and the surrounding fluid is entrained. As a result, two low-pressure regions are created near the Coanda steps on either side of the jet flow. The jet flow is then drawn by one of the low-pressure regions to the left or right attachment wall, creating an attachment flow (Coanda effect).^17^ Due to the expanded dimension downstream of the mixing chamber, the pressure *P*_B_ is greater than *P*_A_, which is near the entrance of the mixing chamber. The pressure difference *P*_B_ − *P*_A_ can drive some of the attachment flow to enter the feedback channel and form an internal circulation (feedback flow). Here, the splitter can further force more fluid to circulate through the feedback channel. At the exit of the feedback channel near the entrance to the mixing chamber, the circulating feedback flow transversely pushes the attachment flow to swing to the opposite side. Subsequently, a new attachment flow is formed on the opposite side. The above phenomenon repeats itself, resulting in a flow oscillation being produced consequently. Here, two fluids are folded, stretched, broken, and merged by the oscillation, and thus they can be efficiently mixed. It has been proved that the oscillation in OFMs can induce intense chaotic convection to achieve high-efficiency micromixing with short residence times^8,18,19^.

### Selective scaling-out

Unlike the parallel scaling mode of the numbering-up strategy, the selective scaling-out strategy is a size expansion method. In this method, the depth of an OFM remains unchanged, but the width and length are linearly increased. Because the oscillation mainly occurs in the width direction and induces chaotic convection, the mixing and mass transfer in this direction are significantly enhanced. Thus, under the condition of chaotic convection, scaling up the width can effectively increase the throughput by reducing the flow resistance without reducing the mixing efficiency in the width direction. The prolonged length can ensure that the residence time (contact time or reaction time) is not greatly reduced due to the increase in throughput. Furthermore, convection in the depth direction of the OFM is much weaker than that in the width direction. Therefore, the depth is not increased and remains at the microscale. The mixing time in the depth direction can be significantly reduced due to the extremely short mixing distance at the microscale. Overall, the selective scaling-out strategy is not a full-dimension scaling method, but a selective-dimension scaling method. Here, the overall mixing and mass transfer efficiency can not be greatly reduced with increasing throughput. The problems caused by the complex microchannel networks and the clogging of NPs in the numbering-up method can be avoided.

**Fig. 2:**
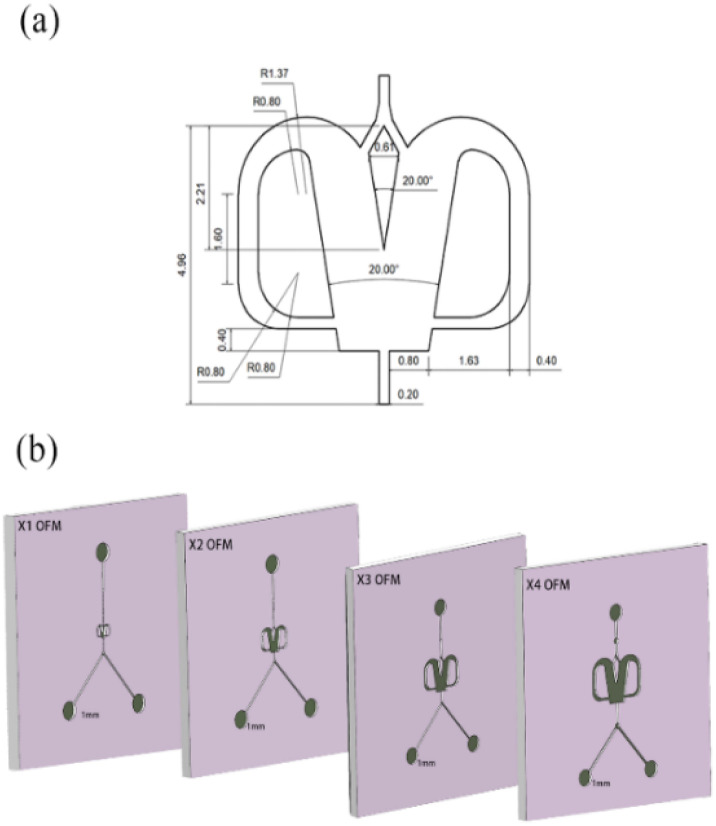
Selective scaling-out strategy: (a) 1X OFM dimensions and (b) scaling-out: from 1X to 4X with an unchanged depth of 1000 μm.

In detail, Fig. 2 describes the selective scale-out of a 1X OFM. The dimensions of the 1X OFM are shown in Fig. 2a. The widths of the entrance and exit channels are both 0.2 mm, the width of the feedback flow channel is 0.4 mm, and the width of the Conda step side is 0.8 mm. The angle between the two attachment walls is 20 degrees. As shown in Fig. 2b, the 1X OFM was linearly scaled out in the width and length directions by 2, 3, and 4 times (enlargement factor, EF), respectively. Meanwhile, the depth of 1000 μm was always kept constant. Each OFM, machined on a main polymethylmethacrylate (PMMA) plate (90 mm × 90 mm × 8 mm) using an LSmicro2020 CNC engraver, allows for flow pattern observation due to the PMMA's transparency. The main plate with microchannels was then bonded to another PMMA plate (90 mm × 90 mm × 8 mm) by thermoforming at 115 °C and 3 bar to form an OFM available for NP synthesis (ESI-1, Fig. S1†).^19^

### Chemicals

BaSO_4_ NPs were used to demonstrate the high-throughput NP synthesis in scaled-out OFMs. Here, sodium chloride dihydrate (BaCl_2_·2H_2_O, AR, Shanghai Meryer Chemical Technology Co., Ltd.), sodium sulfate (Na_2_SO_4_, AR, Shanghai Macklin Biochemical Co., Ltd.), polyethylene (PEG-400, AR, Shanghai Rhawn Co., Ltd.), and anhydrous ethanol (Beijing Tong Guang Fine Chemical Company) were used to produce the BaSO_4_ NPs. A red dye (Qing Ent Company) was used to observe the flow patterns. A blue dye (Qing Ent Company) was used to measure the mixing index.

### Experimental setup and methods

**Fig. 3:**
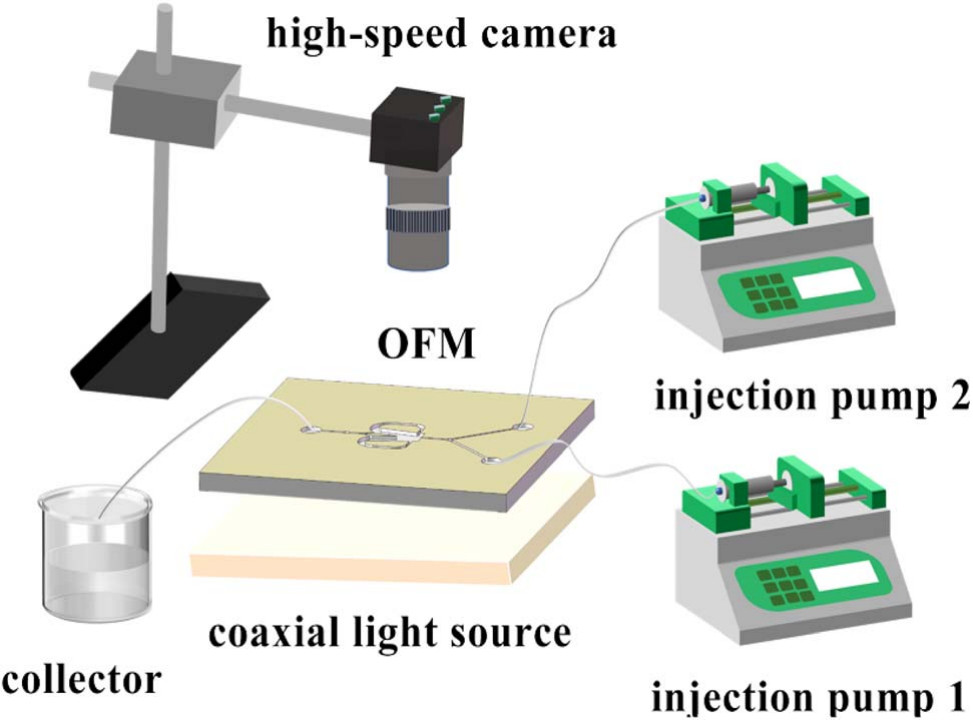
Schematic of experimental setup.

• Flow pattern experiment/the mixing index measurement: As shown in Fig. 3, two phases (colored aqueous phase and 0.1 mol L^−1^ Na_2_SO_4_ aqueous solution) were continuously fed into the Y-shaped inlet channel by two high-precision injection pumps (TGD01), mixed in an OFM, and discharged from the outlet channel into a waste collection container. The red dye was mixed with deionized water in the flow pattern experiment, while the blue dye was mixed with deionized water in the mixing index measurement. A coaxial light source (JK-PWT6024-2) was placed under the OFM with a light diffuser plate. A high-speed camera (1872-ST-160, Germany) was placed above the OFM. The camera was connected to a computer for real-time observation and recording of the flow patterns.

• BaSO_4_ NP synthesis: The experimental setup is the same as that in the flow pattern experiments. Two precursor solutions of BaCl_2_ (0.1–0.3 mol L^−1^) containing 1 wt% PEG (as surfactant) and Na_2_SO_4_ (0.1–0.3 mol L^−1^) were continuously fed into an OFM. The two solutions were mixed while the BaSO_4_ precipitation reaction took place in the OFM. The effluent was collected in a beaker and immediately centrifuged (speed 8000 rpm, 1 min) to separate solid NPs from the liquid phase. The solid NPs were then washed once with water and twice with anhydrous ethanol. Furthermore, the washed NPs were dried in an oven (50 °C, 24 h) to obtain white BaSO_4_ NPs.

• Measurement and characterization methods: a transmission electron microscope (TEM, HT7700 Nippon High-Tech) was used to characterize the morphology of BaSO_4_ NPs. When photographing in the TEM, multiple images in the same condition were taken to ensure randomness. In addition, various images taken were measured separately using Image J software to determine the NP size distribution and the mean size.

## Results and discussion

Re (=*ρud*/*μ*) was used to characterize the flow inside OFMs where *ρ* (kg m^−3^) is the average density of two reactant phases (BaCl_2_ and Na_2_SO_4_ solutions), *u* (m s^−1^) total apparent rate of the two phases at the straight inlet channel (component 4 in Fig. 1), *d* (*m*) the hydrodynamic diameter of the straight inlet channel, and μ (Pa s^−1^) the average kinematic viscosity of the two phases.

### Flow patterns and scaling effects

Flow patterns in OFMs are essential for mixing and mass transfer efficiency. To investigate the flow characteristics in OFMs during the synthesis of BaSO_4_ NPs, a series of flow pattern experiments were first performed in the 1X OFM and recorded by a high-speed camera. Here, deionized water dissolving quantitative red dye (*Q*_R_, 1 g-dye/300 g water) and a colorless Na_2_SO_4_ solution of 0.1 mol L^−1^ containing 20% anhydrous ethanol (*Q*_L_) were fed from the right side and the left side of the Y-channel, respectively. In all experiments, *Q*_R_ and *Q*_L_ were always kept equal and their values were between 0.5 and 28.34 mL min^−1^. Four flow patterns were found and summarised in Table 1. In the laminar pattern, vortex and feedback flow patterns, mass transfer is dominated by low-efficiency molecular diffusion. As a result, mixing is insufficient, and thus uniform concentration field cannot be achieved (ESI-2 and Fig. S2†). This is unfavorable for the synthesis of high-quality nanoparticles. In contrast, the oscillating flow with sufficient circulation (*i.e.*, intense feedback flow) through the feedback channels can disrupt the straight interface, resulting in sufficient mixing. Thus, the oscillating flow pattern is popular for the high-throughput synthesis of NPs because of the efficient and rapid mixing and mass transfer rates with the high throughput.

**Table 1:** Flow patterns at different throughputs (ESI-2, Fig. S2)

*Q*_R_ (mL min^−1^)	Re	Flow patterns	Features	Mixing and mass transfer	
0.5	16.94	Laminar flow	Clear interface without secondary convection	Slight mixing: Molecular diffusion	Fig. S2(a), Video S1
2.0	67.76	Vortex flow and feedback flow	Clear interface, vortex, and internal circulation	Partial mixing: Molecular diffusion (main) and convection (auxiliary)	Fig. S2(b) and (c), Videos S2 and S3
5.0	169.4				
12.5	423.5	Oscillating flow	Intense oscillation and sufficient circulation	Sufficient mixing: Convection	Fig. S2(d)–(f), Videos S4 and S5

**Fig. 4:**
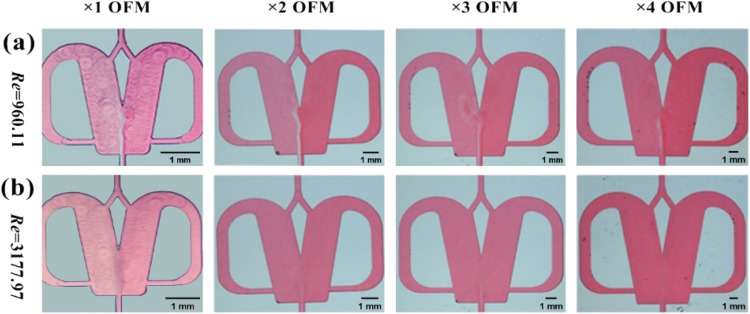
Effects of the enlargement factor EF and Reynolds number on oscillating flow pattern in OFMs (images of OFMs with different EFs are scaled to the same size): (a) Re = 960.1 and (b) Re = 3178. For comparison, the views of OFMs with different EFs are scaled approximately to the same size.

The effects of the scale-up on the oscillating flow pattern were further investigated to determine the feasibility of scaled-out OFMs for the high-throughput synthesis of NPs (see ESI-3† for detailed information about scaling effects on all flow patterns; Fig. S3 and Videos S6– S21†). As shown in Fig. 4(a) (Videos S14–S17†), the 1X–4X OFMs all produce an intense oscillating flow at Re of 960.1. The two phases are mixed relatively uniformly in the mixing chamber, but there is still a significant color difference between the left and right channel. In the 4X OFM, the oscillation frequency is lower than that of the 1X OFM, indicating a relatively poorer mixing performance. It can be seen that the depth of the red color on the left side of the 4X OFM is a little lighter than that on its right side. When the Re number is further increased to 3178, the colors of the two phases in the 1X–4X OFMs are almost identical, as shown in Fig. 4(d) (Videos S18–S21†). Here, complete mixing is achieved where the concentration is uniform everywhere and the residence time is rather short due to the high rate at such a high Re. The uniform concentration field and short residence time are expected for the synthesis of high-quality and high-productivity NPs. At sufficiently high Re numbers, the scaling effects of the scaled-out OFMs can be neglected, making these OFMs the ideal NP synthesizers.

### Evaluation of mixing performance

To quantify the mixing performance in the 1X–4X OFMs at different Re numbers, flow pattern images were processed with Matlab. A mixing index (X) was used to evaluate mixing performance^9^ as illustrated in eqn (1)–(3). The normalized gray value *I*_*i*,*n*_ was calculated using eqn (1), in which *I*_*i*_, *I*_max_ and *I*_min_ are the gray value (0–255) of pixel *I* and the maximum and the minimum gray values in the selected region of an OFM, respectively. Eqn (2) describes the standard deviation of the grayscale value of pixel *i*, where *I*_*i*,*n*_ is the normalized grayscale value, 
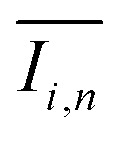
 is the average of all *I*_*i*,*n*_, and *N* is the number of pixels. Eqn (3) calculates the mixing index *X* in the selected region, where *σ*_cm_ and *σ*_um_ are the standard deviation of the complete mixing and non-mixing in the OFM, respectively.1
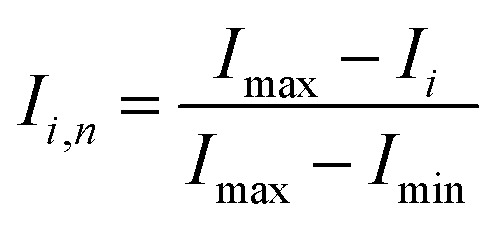
2
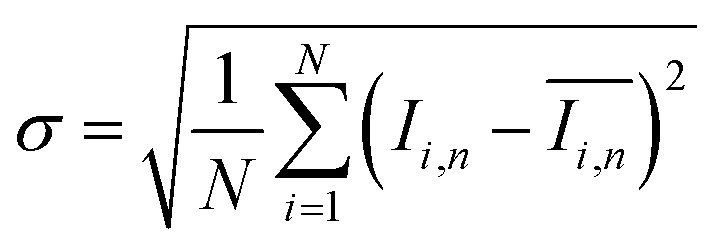
3
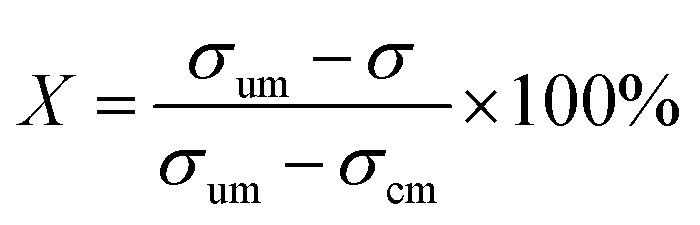


**Fig. 5:**
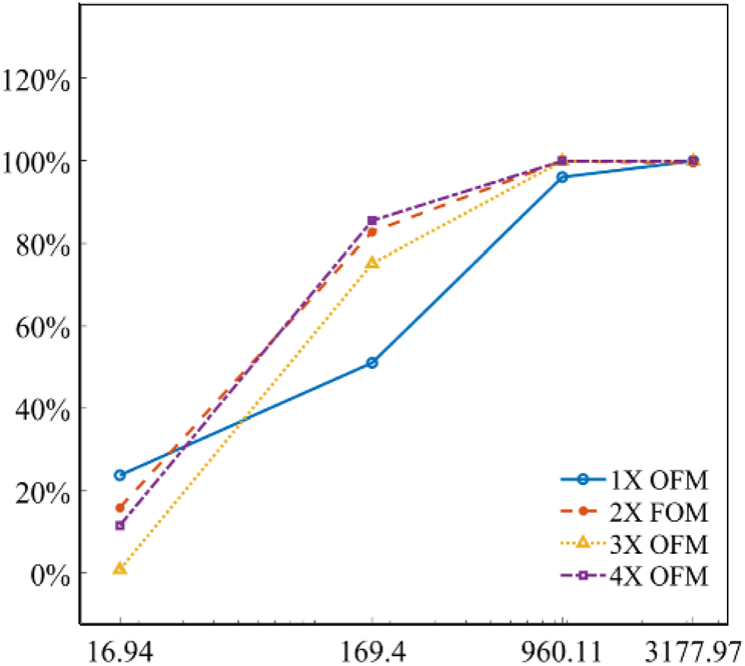
Mixing indexes of 1X–4X OFMs at different Re numbers.

Fig. 5 describes the mixing index of 1X–4X OFMs at different Re numbers (from 16.94 to 3177.97). The mixing indexes increased with the rise of Re in 1X–4X OFMs. When Re was 16.94 and 169.4, the mixing indexes of the 3X OFM and 4X OFM were smaller than that of the 1X OFM and 2X OFM. This indicates that the enlarged OFMs have worse mixing performance under lower Re numbers. As Re increased to 960.11, the mixing indexes of 1X–4X OFMs were all above 90%. When Re was up to 3177.97, the mixing indexes of 1X–4X OFMs were nearly 100%. This indicates that as long as Re is high enough, 1X–4X OFMs can achieve complete mixing.

### Nanoparticle synthesis

As mentioned above, different flow patterns can significantly affect the mixing and mass transfer performance, and thus determine the quality of NPs synthesized in scaled-out OFMs. To demonstrate the feasibility of high-throughput NP synthesis, BaSO_4_ NPs were synthesized using scaled-out OFMs in this section and two synthesis modes were found.

#### Non-chaotic convection synthesis

Laminar flow and secondary flows such as the vortex and feedback flow patterns were first investigated and an interesting synthesis mode, interface synthesis, was found. Here, a BaCl_2_ solution (0.1 M, throughput *Q*_R_) and a Na_2_SO_4_ solution (0.1 M, throughput *Q*_L_) were injected into the 2X OFM through the Y-channel inlet at *Q*_R_ = *Q*_L_. In addition, to better observe the flow patterns during NP synthesis, a certain amount of red dye (1 g-dye/300 g water) was added to the BaCl_2_ solution, which can easily identify the positions of NP generation. Corresponding flow patterns without NP synthesis were also recorded for comparison.

**Fig. 6:**
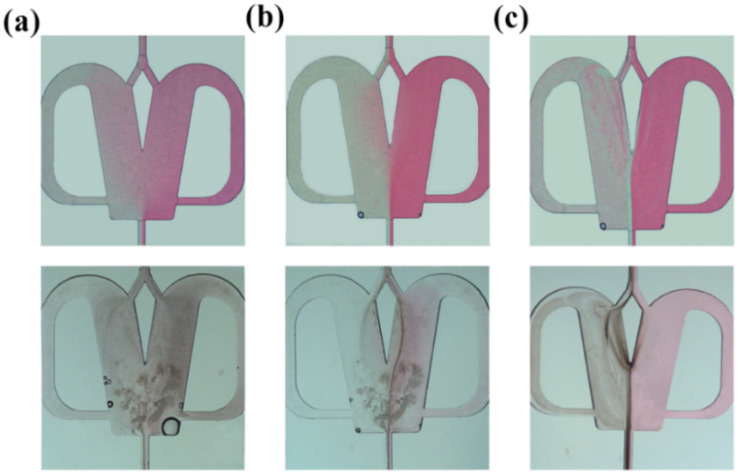
Non-chaotic convection synthesis dominated by molecular diffusion in 2X OFM: (a) *Q*_R_ = 0.07 mL min^−1^; (b) *Q*_R_ = 0.7 mL min^−1^; (c) *Q*_R_ = 7 mL min^−1^ (top: flow pattern without NP synthesis, bottom: flow pattern with NP synthesis).

• Laminar flow synthesis: the experimental results with *Q*_R_ = *Q*_L_ = 0.07–7 mL min^−1^ are shown in Fig. 6. In Fig. 6(a) (Videos S22 and S25†), a laminar flow with non-uniform diffusion mixing could be seen (top). The bottom flow pattern with NP synthesis also showed that BaSO_4_ NPs were formed only in the tracer dye spreading region. In this case, the concentrations of BaCl_2_ and Na_2_SO_4_ were non-uniform and the residence time was long due to the low throughput. Thus, the generated BaSO_4_ NPs tended to overgrow and agglomerate, forming a wide particle size distribution. A similar phenomenon could be found in reported works using other synthesizers.^19,20^Fig. 6(b) shows the laminar flow at the throughput of 0.7 mL min^−1^. The phenomenon was similar to Fig. 6(a), but there was a back-and-forth circulation of the fluid at the interface near the splitter tip (Videos S23 and S26†). The circulation phenomenon was more evident in the flow pattern with NP synthesis (bottom of Fig. 6(b)). Moreover, a clear interface formed by the generated NPs can also be seen. This was an interfacial synthesis phenomenon. Here, the reaction rate was faster than the low-efficiency diffusion mixing rate, and thus the BaCl_2_ and Na_2_SO_4_ couldn't diffuse into their opposite bulk phase. Consequently, many NPs were formed only at the interface between the BaCl_2_ and Na_2_SO_4_ solutions.

• Vortex and feedback flow synthesis: the laminar flow was still dominant at *Q*_R_ = *Q*_L_ = 7 mL min^−1^ (Fig. 6(c) and Video S24†), but the secondary flows, *i.e.*, the vortex and feedback flows were also evident (top of Fig. 6(c)). These secondary flows which are transversed to the main laminar flow relatively enhance the mixing and mass transfer performance.^21^ It could be seen in the bottom of Fig. 6(c) that the generated NPs appeared throughout the micromixer, but an interface formed by the generated NPs was still noticeable. Thus, interface synthesis was still a dominant mode in this case. In addition, it was also worth noting that the feedback flow in the feedback channel was slower than that in the mixing chamber, resulting in a longer residence time of the NPs in the feedback channel and leading to noticeable NP growth. The increasing particle size and agglomerative growth of the BaSO_4_ NPs in the feedback channel could be observed in Video S27.† Therefore, this flow mode was also not suitable for the high-throughput synthesis of high-quality NPs.

**Fig. 7:**
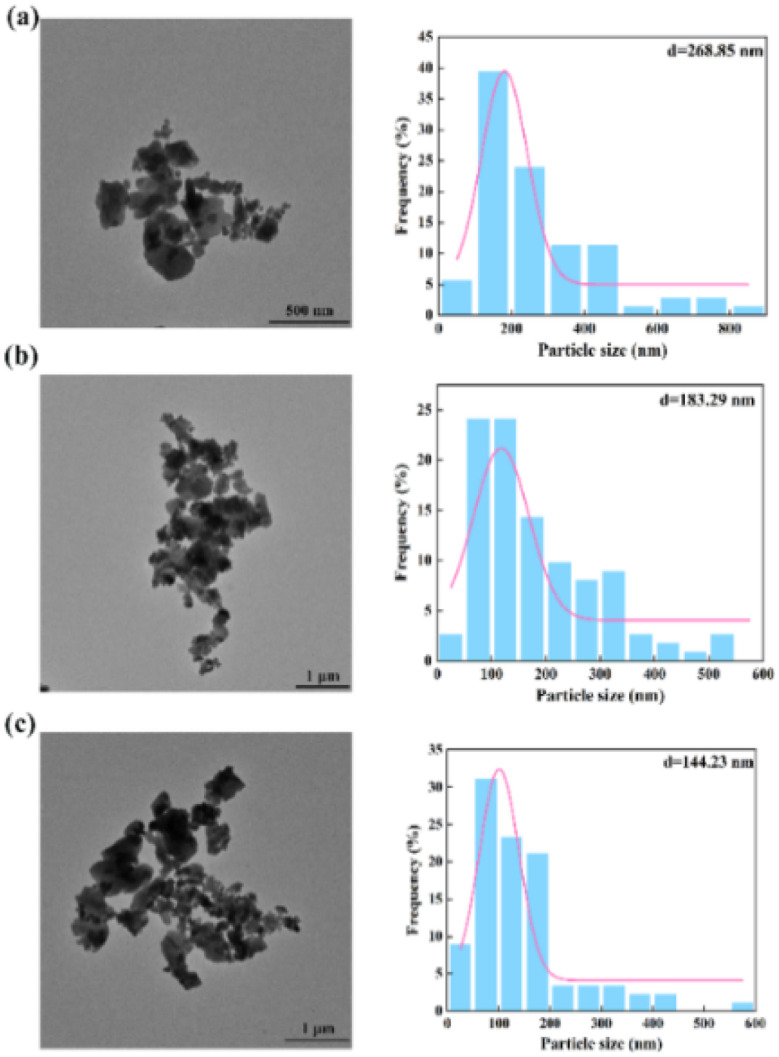
Morphology and size distribution of NPs by non-chaotic convection synthesis: (a) *Q*_R_ = 0.07 mL min^−1^; (b) *Q*_R_ = 0.7 mL min^−1^; (c) *Q*_R_ = 7 mL min^−1^ (Table S2†).

To further verify the above conclusions, Fig. 7 shows the morphology and particle size distribution of Ba_2_SO_4_ NPs which were generated under the same conditions as in Fig. 6 (Table S2†). Consistent with the results of Fig. 6(a), the NPs that were generated in the laminar flow mode were seriously agglomerated (Fig. 7(a)). The mean particle size can reach 268.85 nm with a wide range of particle size distribution. Fig. 7(b) shows slightly less agglomeration than Fig. 7(a). When the throughput was increased to 7 mL min^−1^ (Fig. 7(c)), the mean size of the Ba_2_SO_4_ NPs further decreased, and the particle size distribution became relatively narrow. However, the long residence time in the feedback channel led to NP growth and NP agglomeration was still evident.^22^

In summary, when the throughput is not high enough, scaled-out OFMs are in non-chaotic convection mode. Even though there are some secondary flows such as the vortex and feedback flows, the laminar flow is still dominant. Due to the low efficiency of molecular diffusion, the Ba_2_SO_4_ NPs are mainly formed at the interface between the BaCl_2_ and Na_2_SO_4_ solutions. Moreover, the resulting NPs tend to agglomerate and grow due to the non-uniform concentration field and long residence time.

#### Chaotic convection synthesis

**Fig. 8:**
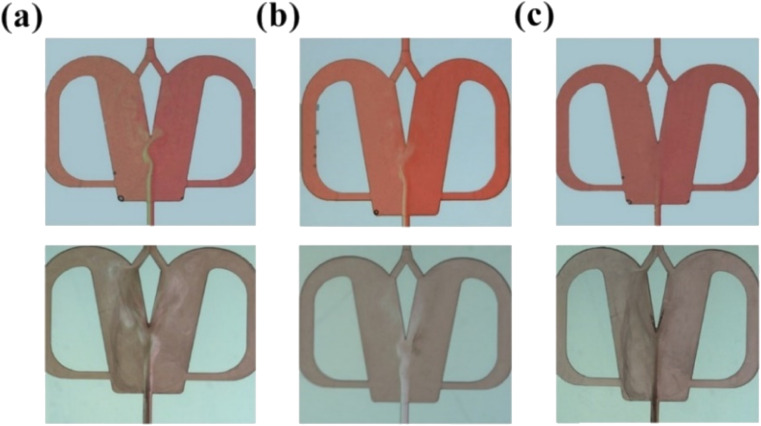
Chaotic convection synthesis (a) *Q*_R_ = 17 mL min^−1^, (b) *Q*_R_ = 33 mL min^−1^, and (c) *Q*_R_ = 70 mL min^−1^ (top: flow pattern without NP synthesis, bottom: flow pattern with NP synthesis).

The flow patterns of chaotic convection with and without NP synthesis in the 2X OFM are shown in Fig. 8. The throughput *Q*_R_ (*Q*_R_ = *Q*_L_) was in the range of 17–70 mL min^−1^ and the chaotic convection was achieved. As can be seen in the upper images of Fig. 8 (Videos S28–S30†), the oscillation became more intense, and thus the color depth over the whole OFM became more uniform with increasing throughput. The flow patterns corresponding with NP synthesis also showed that the BaCl_2_ and Na_2_SO_4_ solutions underwent an intense oscillation and were vigorously mixed, resulting in an extremely uniform concentration field (bottom of Fig. 8 and Videos S31–S33†). During the precipitation reaction, the contact of the two reactants BaCl_2_ and Na_2_SO_4_ resulted in the formation of many homogeneous grains. In the OFM, the very short residence time which was caused by the high throughput effectively limited the grain growth time, and more grains were nucleated without overgrowth, resulting in a smaller NP size and narrow size distribution.

**Fig. 9:**
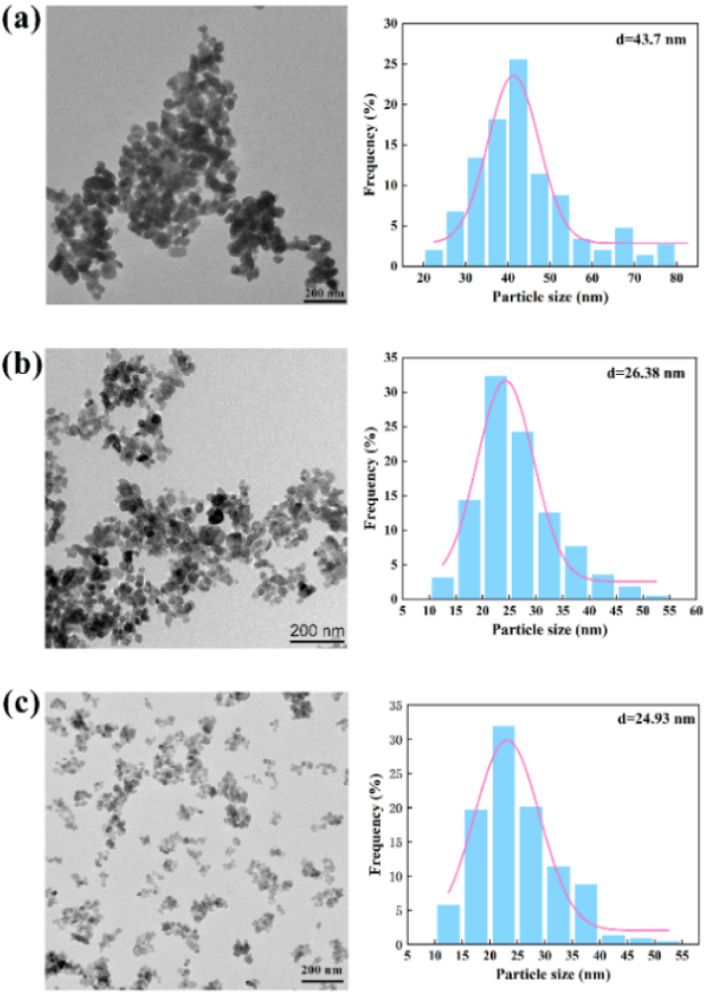
Morphology (left) and size distribution (right) of NPs by chaotic convection synthesis: (a) *Q*_R_ = 17 mL min^−1^; (b) *Q*_R_ = 33.06 mL min^−1^; (c) *Q*_R_ = 70 mL min^−1^ (Table S2†).

Fig. 9 shows the morphology and particle size distribution (Table S2†) of the BaSO_4_ NPs produced by the chaotic convection synthesis. Compared with the non-chaotic convection synthesis, the mean NP size of the chaotic convection synthesis dramatically decreased to several tens of nanometers. Moreover, the mean NP size decreased as the throughput increased and its size distribution became more concentrated. The agglomeration of the generated NPs was significantly improved by increasing the throughput *Q*_R_ from 17 mL min^−1^ to 70 mL min^−1^. In particular, the agglomeration diminished and the minimum size of the synthesized BaSO_4_ NPs was about 10 nm at the throughput of 70 mL min^−1^. This phenomenon was inspiring because the scaled-out OFM enabled the high-throughput and high-quality synthesis of NPs.

To further analyze the differences between the non-chaotic and chaotic convection synthesis, the NP size distributions using the 2X OFM at different throughputs are shown in Table 2. It could be seen that as the throughput increased, the residence time reduced and consequently the mean size also decreased, which was similar to Chen *et al.*'s^23^ experimental conclusions. It could be confirmed that chaotic convection significantly reduced the NP size compared to non-chaotic convection. Moreover, in the chaotic convection mode, the larger the throughput, the smaller the NP size. Thus it could be demonstrated that the chaotic convection mode induced by high throughput could provide a uniform concentration field and very short residence time, which was essential for the large-scale production of high-quality NPs. Therefore, it could be confirmed that passive scaled-out OFMs were the ideal candidate synthesizers to generate intense chaotic convection for NP production.

**Table 2:** Comparison of NP size distribution under different throughputs (2X OFM)

Synthesis mode	Non-chaotic			Chaotic		
Total throughput (mL min^−1^)	0.14	1.4	14	34	66.12	140
Residence time (s)	24.10	2.41	0.24	0.10	0.05	0.02
Mean NP size (nm)	268.85	183.29	144.23	43.7	26.38	24.93

#### Effects of feedback channels on chaotic convection synthesis

In previous studies, a coaxial turbulent jet mixer which was similar to OFMs without the feedback channels has been recognized for its simple structure, high throughput, and ability to synthesize high-quality nanoparticles.^24^ To validate the effects of the feedback channels, a 2X OFM without the feedback channels as shown in Fig. S4 (ESI-4†) was also used to synthesize BaSO_4_ NPs under chaotic convection (total throughput of 34–140 mL min^−1^). Table 3 compares the properties of BaSO_4_ NPs synthesized in 2X OFMs with and without feedback channels. It could be confirmed that the OFM with the feedback channels could produce smaller NPs. This was because the feedback channels facilitated fluid mixing and mass transfer through internal recirculation flow and fluid oscillation.

**Table 3:** Effects of feedback channels on chaotic convection synthesis

Total throughput (mL min^−1^)	Mean NP size in 2X OFM (nm)	
	With feedback channels	Without feedback channels
34	43.7	88.4
66.12	26.38	62.6
140	24.93	54.3

#### Performance verification: high-throughput synthesis of high-quality NPs

Large-scale and high-quality synthesis of NPs is the goal of this work. For this purpose, the 2X–4X OFMs in the chaotic convection mode (Re = 1920 and 3178) were systematically investigated to verify their performance in the high-throughput and high-productivity synthesis of BaSO_4_ NPs.

**Fig. 10:**
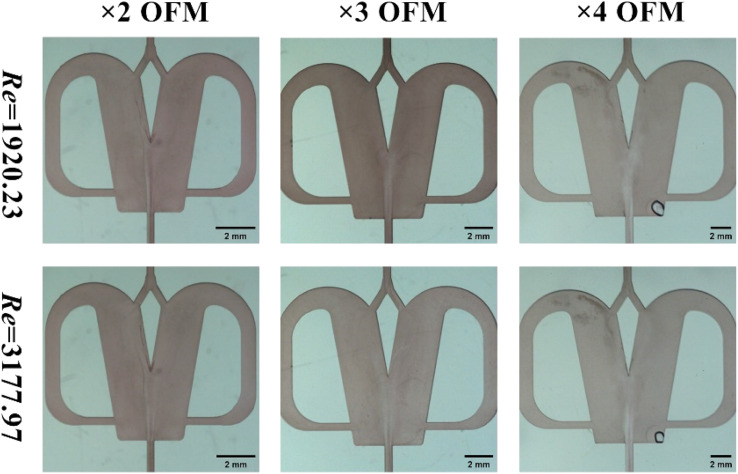
Flow patterns with chaotic convection synthesis of BaSO_4_ NPs in 2X–4X OFMs (Re = 1920 and Re = 3178, 0.1 mol L^−1^ BaCl_2_ + 0.1 mol L^−1^ Na_2_SO_4_).

**Fig. 11:**
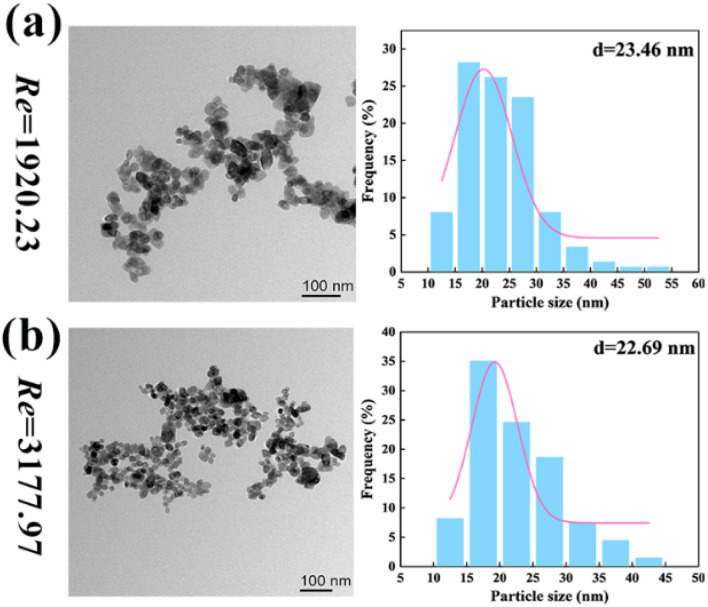
Morphologies and size distributions of NPs by chaotic convection synthesis in 4X OFMs ((a) Re = 1920 and (b) Re = 3178, reactants: 0.1 mol L^−1^ BaCl_2_ + 0.1 mol L^−1^ Na_2_SO_4_; Table S2†).

As shown in Fig. 10 (reactants: 0.1 mol L^−1^ BaCl_2_ + 0.1 mol L^−1^ Na_2_SO_4_; Videos S34–S39†), the BaSO_4_ NPs which were generated at such a high Re number were so fine that they were almost invisible. In Table 4, the corresponding residence times at Re = 1920 and 3178 were just 0.0154 s–0.077 s. The extremely small residence time could effectively control the further growth of nucleated BaSO_4_ NPs, producing a larger number of tiny NPs. Moreover, the BaSO_4_ NP growth in these OFMs was also hindered because there was almost no NP retention due to the high throughput. Furthermore, the high throughput was also impressive. The total throughput (*i.e.*, *Q*_R_ + *Q*_L_) of the 4X OFM can reach 281.4 mL min^−1^, which is much higher than the maximum value of 160 mL min^−1^ reported in ref. 11. The morphology and particle size distribution of the generated BaSO_4_ NPs in the 4X OFM are shown in Fig. 11 (Table S2†). It could be seen that the Re of up to 1920 and 3178 did not affect the particle size and its distribution. Moreover, the particle size of the BaSO_4_ NPs was almost regulated within a narrow range of 10–55 nm. The mean size was only 22.69 nm. Overall, high-quality BaSO_4_ NPs could be obtained at such a high throughput of 281.4 mL min^−1^ using the 4X OFM. Meanwhile, the BaSO_4_ NP production rate could reach 179.4 g h^−1^ at a concentration of 0.1 mol L^−1^ for both reactants BaCl_2_ and Na_2_SO_4_.

**Table 4:** High-throughput synthesis of BaSO_4_ NPs in chaotic convection mode

EF		2X	3X	4X
Re = 1920	Total throughput (mL min^−1^)	132.24	151.14	170.04
	Residence time (s)	0.0255	0.049	0.077
Re = 3178	Total throughput (mL min^−1^)	218.86	250.14	281.4
	Residence time (s)	0.0154	0.030	0.047

To significantly increase the NP production rate, the BaCl_2_ and Na_2_SO_4_ concentrations were further increased from 0.1 mol L^−1^ to 0.15–0.30 mol L^−1^, and the results are shown in Table 5. It could be found that all NP yields exceeded 90% (ESI-5†). Importantly, the BaSO_4_ NP production rate increased with increasing reactant concentration. When the BaCl_2_ and Na_2_SO_4_ concentrations reached 0.3 mol L^−1^, an NP production rate of 538.4 g h^−1^ could be obtained. This value was much greater than the maximum value of 105.5 g h^−1^ reported. In addition, it could also be seen that the reactant concentration has less effect on the NP size, and all mean NP sizes remain in the range of 20–30 nm (Fig. S5†). Thus, the 4X OFM was shown to enable high-quality and high-productivity production of BaSO_4_ NPs.

**Table 5:** The production rates and mean size of nanoparticles in 4X OFM with different concentrations (total throughput 281.4 mL min^−1^ and Re = 3178)

BaCl_2_ and Na_2_SO_4_ (moL L^−1^)	Yield	Production rate (g h^−1^)	Mean NP size (nm)
0.1	91.2%	179.4	22.69
0.15	91.93%	271.2	23.66
0.2	93.28%	367.0	25.36
0.25	90.53%	445.20	26.15
0.3	91.24%	538.4	24.91

## Conclusions

In this work, BaSO_4_ NPs were fabricated by using selectively scaled-out OFMs to demonstrate the feasibility of high-throughput and high-quality NP synthesis. It can be confirmed that chaotic convection is essential for the high-quality synthesis of NPs using scaled-out OFMs. The non-chaotic convection mode such as the laminar flow, vortex flow, and feedback flow is not suitable for the high-throughput and high-quality synthesis of NPs. In chaotic convection at high enough Reynold numbers (Re = 960.1–3178), the mixing performance is not affected by the scaling size. The chaotic convection mode can effectively control the growth of the nucleated NPs due to the very short residence time and uniform concentration field, and thus high-quality NPs with narrow size distribution and small mean size can be obtained at high throughput. The 4X OFM can be operated at a high total throughput of up to 281.4 mL min^−1^. High BaSO_4_ NP production rates of up to 538.4 g h^−1^ were obtained. Meanwhile, the NP mean size is between 20 and 30 nm, and the NP size distribution is just in the narrow range of 10 nm ∼50 nm. The selective scaling-out strategy was confirmed to be effective in significantly increasing the throughput of OFMs, providing a feasible way for the high-throughput and high-productivity synthesis of high-quality NPs.

## Author contributions

Cong Xu designed the research and reviewed the paper; Mingxin Li conducted the experiments, analyzed the data, and wrote the paper; Wensheng Wang partly conducted the experiments and analyzed the data.

## Conflicts of interest

The authors declare that they have no known competing financial interests or personal relationships that could have appeared to influence the work reported in this paper.

## Supplementary Material

RA-013-D3RA08037D-s001

RA-013-D3RA08037D-s002

RA-013-D3RA08037D-s003

RA-013-D3RA08037D-s004

RA-013-D3RA08037D-s005

RA-013-D3RA08037D-s006

RA-013-D3RA08037D-s007

RA-013-D3RA08037D-s008

RA-013-D3RA08037D-s009

RA-013-D3RA08037D-s010
