# Supplementary material for: High-throughput synthesis of nanoparticles using oscillating feedback microreactors: a selective scaling-out strategy
Source: RSC Adv. 2023 Dec 11;13(50):35435–44. doi: 10.1039/d3ra08037d (PMC10711568; doi:10.1039/d3ra08037d)
Supplement: RA-013-D3RA08037D-s001 [file RA-013-D3RA08037D-s001.pdf]

Electronic Supplementary Material (ESI) for RSC Advances.
This journal is © The Royal Society of Chemistry 2023

Supplementary Information

High-Throughput Synthesis of Nanoparticles Using Oscillating Feedback Microreactor: Selective Scaling-out Strategy

Mingxin Li, [a] Wensheng Wang [a] and Cong Xu [a]*

[a] *Institute of Nuclear and New Energy Technology, Tsinghua University, Beijing, 100084, P.R China*

Corresponding Author:

Cong Xu

Institute of Nuclear and New Energy Technology, Tsinghua University

Chengfu Road, Haidian District, Beijing

P. O. Box 1021, Postcode 102201

People's Republic of China

Tel: 86- 10- 89796075, Fax: 86-10- 62771740, E-mail: c-xu@mail.tsinghua.edu.cn

Co-author:

Mingxin Li, Wensheng Wang

Institute of Nuclear and New Energy Technology, Tsinghua University

Chengfu Road, Haidian District, Beijing

P. O. Box 1021, Postcode 102201

People's Republic of China

Tel: 86- 10- 89796222, Fax: 86-10- 62771740

Email: limx21@mails.tsinghua.edu.cn; wws19@mails.tsinghua.edu.cn

SI-1 Oscillating Feedback Microreactor Fabrication

As shown in Fig. S1, the LSmicro2020 CNC engraving machine was used to carve the flow channel on a transparent PMMA plate with a processing speed of 22000 rpm, a feed rate of 35 mm/min, and a processing time of 2 h. Then the carved plate and a smooth cover plate were cleaned ultrasonically several times. After that, the two PMMA plates were placed in a vacuum-drying oven at a temperature of 80℃ for 30 min. The surfaces of the two dried PMMA plates were further treated in plasma with a plasma power of 170 W, vacuum pressure of 30mTorr, and treatment time of 30 s. Then, the carved plate and the cover plate were immediately stacked together in the vacuum heat press LSmicro-nanoprint 100 for hot pressing bonding. The heating rate is 9℃/min, the hot pressing temperature is 115℃, the hot pressing pressure is 3 Bar, and the hot pressing time is 33 min. After hot pressing, the temperature was decreased at a cooling rate of 5℃/min. Finally, the pressing device was removed when the temperature was lowered to below 45℃, and a complete OFM was obtained.

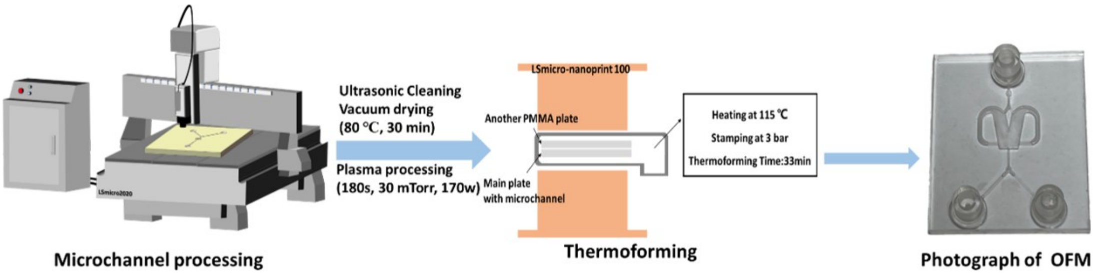

Fig. S1 Manufacturing process diagram of OFM

SI-2 Flow Pattern Classification

Flow patterns in OFMs are essential for mixing and mass transfer efficiency. To

investigate the flow characteristics in OFMs during the synthesis of $BaSO_4$ NPs, a series of flow pattern experiments were performed in the 1X OFM. As shown in Fig. S2, deionized water dissolving quantitative red dye was fed into the OFM from the right sides of the Y-channel, and its throughput was defined as Q_R. A colorless Na_2SO_4 solution of 0.1 mol/L containing 20% anhydrous ethanol was fed from the left side of the Y-channel and its throughput was defined as Q_L. In all experiments, Q_R and Q_L were always kept equal and their values were between 0.5-28.34 mL/min in the flow pattern experiments. The flow patterns inside the 1X OFM were recorded by the high-speed camera.

- Laminar flow: Fig. S2(a) (Video S1) showed an ordered flow pattern at a very low throughput of Q_R=0.5 mL/min (*Re*=16.94). A clear interface between the red and colorless liquids could be found, which was a typical feature of laminar flow. The two liquids pass directly through the OFM without any convection transverse to the main flow. And there was no feedback flow circulating through the feedback channels. In this case, it was predictable that NP synthesis was dominated by low-efficiency molecular diffusion, and the precipitation reaction was concentrated at the interface between the two liquids [1].

- Vortex flow and feedback flow: As shown in Fig. S2(b) (Video S2), there was still a clear interface between the two phases as Q_R increased to 2.0 mL/min (*Re*=67.76), but obvious secondary flows appeared. An obvious counterclockwise vortex formed on the colorless left side. Moreover, some red liquid entered the feedback channel and

slowly circulated back to the mixing chamber. Such secondary flows of the vortex and the feedback flow became more noticeable when the Q_R was further increased to 5.0 mL/min (Fig. S2(c) and Video S3) (*Re*=169.4). This case indicated that NPs could also be synthesized in the bulk phase of the two liquids, not only at the interface. The secondary flow, i.e., the convection transverse to the main flow could promote the mixing and mass transfer, which was beneficial for NP synthesis. However, the clear and straight interface throughout the whole OFM indicated that low-efficiency molecular diffusion was still a dominant factor for NP synthesis.

- Oscillating flow: When Q_R was increased to 12.5 mL/min (*Re*=423.5), an oscillation occurred, as shown in Fig. S2(d) and Fig. S2(e) (Video S4). In Fig. S2(d), the interface deviated to the right side and immediately began to deviate to the left (Fig. S2(e)), which produced an oscillation. Fig. S2(f) (Video S5) showed a more intense oscillating flow at Q_R=28.3 mL/min (*Re*=960.1). The straight interface throughout the OFM was disrupted and feedback flows circulated rapidly through the feedback channels. In this case, the colors of all liquids in the OFM were essentially identical, indicating a sufficient mixing between the red and colorless liquids. The oscillating flow was popular for the high-throughput synthesis of NPs because of the efficient and rapid mixing and mass transfer rates with the high throughput [2].

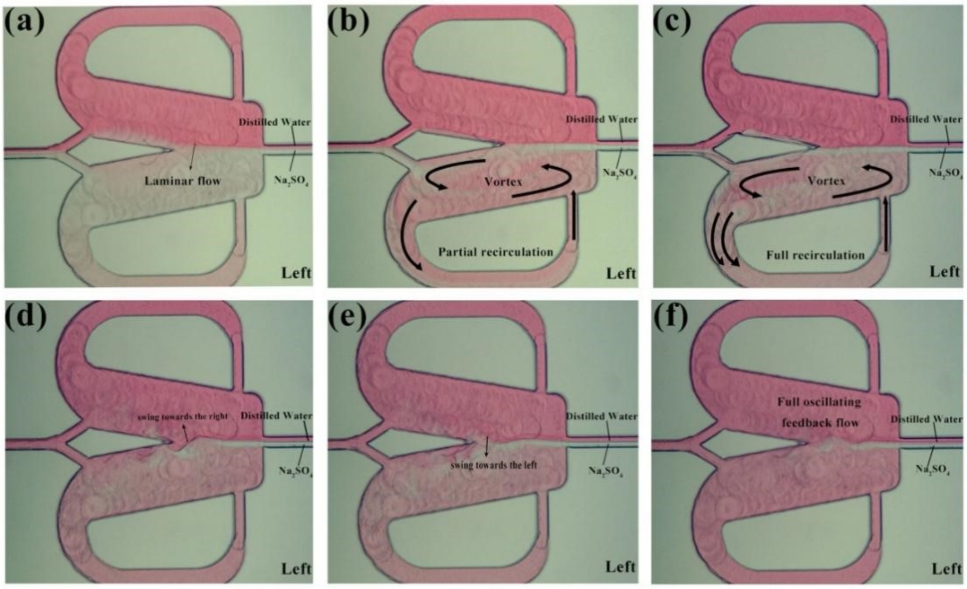

Fig. S2 Flow patterns in 1X OFM (Q_R=Q_L): (a) Q_R=0.5 mL/min; (b) Q_R=2.0 mL/min; (c) Q_R= 5.0 mL/min; (d-e) Q_R=12.5 mL/min; (f) Q_R= 28.3 mL/min

SI-3 Flow Patterns: Scaling Effects

The flow patterns essentially affect the mixing performance. Therefore, the effects of the scale-up on flow patterns were further investigated to determine the feasibility of scaled-out OFMs for high-throughput synthesis of NPs. All experimental conditions were the same as those described in Section SI-2 “Flow Pattern Classification”. The laminar, vortex, and oscillating flows were also observed in the 2X～4X OFMs, but the scaling effects were noticeable.

- Laminar flow: As shown in Fig. S3(a) (Video S6- Video S9), the 1X～4X OFMs were all in laminar flow at *Re*=16.94. However, the mixing performance was affected by the enlargement ratio. In the 2X OFM～4X OFMs, the red deionized water phase diffused into the colorless Na_2SO_4 phase. It could be concluded that the mass that transferred from the red water to the colorless Na_2SO_4 phase was dominated by low-efficiency

molecular diffusion since no secondary flow such as the vortex was observed. Furthermore, the larger the EF of the OFM, the larger the red water phase dispersion. This was because a long residence time was critical for increasing the diffusion mixing efficiency. This non-uniform mixing was not conducive to producing high-quality NPs with narrow particle size distribution, and therefore the laminar flow mode should be avoided in scaled-out OFMs.

- Vortex and feedback flow: As shown in Fig. S3(b) (Video S10- Video S13), the secondary flows such as vortex and partial feedback flows occurred in the 1X OFM at *Re* = 67.76, but the 4X OFM still maintained the laminar flow with a rather clear interface throughout the whole OFM. As for the 2X OFM, the vortex flow was only observed in the mixing chamber. When the OFM size was increased to 3X, there was no vortex and feedback flow to be observed. Compared with the 1X OFM, the mixing performance of the 2X～4X OFMs was inferior. Although the *Re* was the same, the velocity in the scaled-out OFMs was lower than that in the 1X OFM. The larger the size of the OFM, the lower the velocity near the exit of the OFM. Consequently, the mixing chamber could not generate sufficient pressure difference between both ends of the feedback channel to form the recirculating flow. The lower pressure difference between the Coanda step and the exit of the mixing chamber also could not generate a vortex. Therefore, the 2X～4X OFMs could not have excellent mixing performance.
- Circulating flow: As shown in Fig. S3(c) (Video S14- Video S17)**,** the 1X～4X OFMs all generated an intense oscillating flow at *Re* of 960.1. The two phases were mixed

relatively uniformly in the mixing chamber, but there was still a significant color difference between the left and right channels. In the 4X OFM, the oscillation frequency was lower than that of the 1X OFM, indicating a relatively poorer mixing performance. It could be seen that the depth of the red color on the left side of the 4X OFM was a little lighter than that on its right side. When the *Re* number was further increased to 3178, the colors of the two phases in the 1X～4X OFMs were almost identical, as shown in Fig. S3(d) (Video S18- Video S21). Here, complete mixing was achieved where the concentration was uniform everywhere and the residence time was rather short due to the high velocity at such high *Re*. The uniform concentration field and short residence time were essential for the synthesis of high-quality NPs. In this case, the scaling effects of the scaled-out OFMs could be neglected, making these OFMs ideal NP synthesizers.

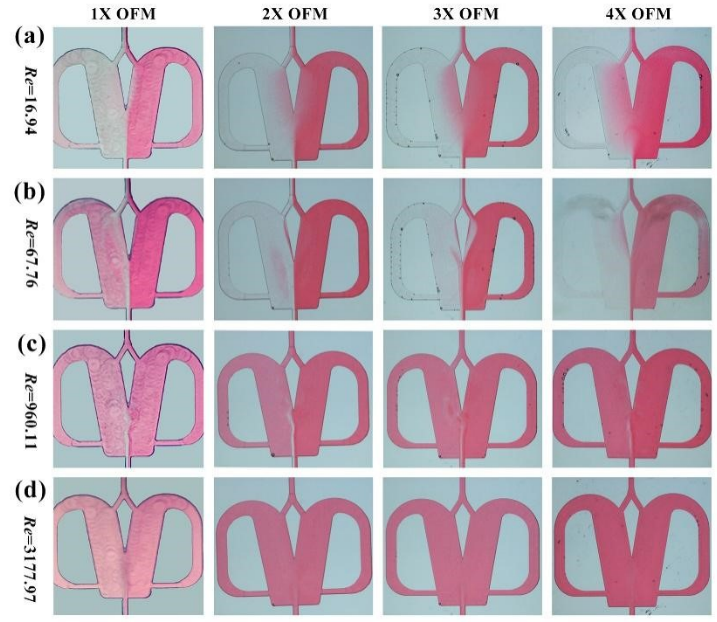

Fig. S3 Effects of the enlargement factor EF and Reynolds number on flow patterns in OFMs: (a) *Re*=16.94; (b) *Re*=67.76; (c) *Re*=960.1; (d) *Re*=3178

SI-4 OFM without feedback channels

Fig. S4 shows the structure of the 2X OFM without feedback channels. Except for the feedback channels, the other structure is the same as the 2X OFM.

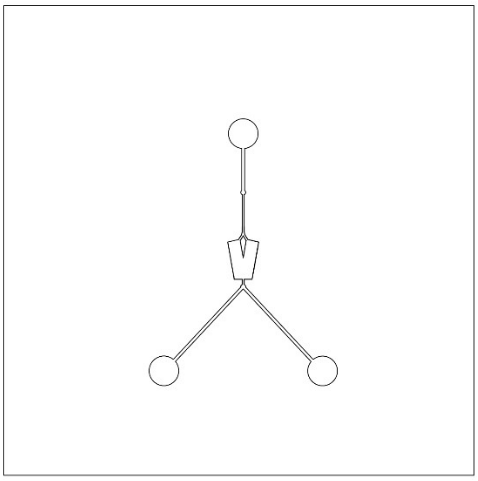

Fig. S4 OFM without feedback channels

SI-5 Measurement of $BaSO_4$ NP Production Rate and Yield

As shown in Fig. S5, set the concentration ratio of $BaCl_2$ and Na_2SO_4 to maintain 1:1 at all times and change the concentrations to 0.1, 0.15, 0.2, 0.25, and 0.3 moL/L. The flow rates of the two phases were both 140.7 mL/min (the total flow rate was 281.4 mL/min), and the volumes of $BaCl_2$ and Na_2SO_4 solutions were both 10 mL (the total volume of reactant solutions, V_{total}=20mL). The two reactant solutions were fed into the 4X OFM. A bottle containing 200 mL of deionized water was placed at the outlet of the 4X OFM to collect NPs. The amount of deionized water was much higher than the amount of reactants so the unreacted reactants were sufficiently diluted in the collection bottle. As a result, the reaction between unreacted $BaCl_2$ and Na_2SO_4 coming out of the OFM could be neglected. Namely, the NP synthesis reaction could

be quenched in the collection bottle so as not to affect the yield and production rate measurement of NPs generated within the OFM. Then, the collected product solution in the bottle was immediately centrifuged (8000 rpm, 1 min) to obtain wet $BaSO_4$ NPs (Quality M_c, g). The collected NPs were washed once with water and twice with anhydrous ethanol, and the wet NPs were further dried to obtain white NPs (50 °C, 24 h). As a result, the actual NP production rate and yield could be determined by the following equations.

$$BaSO_4 \text{ NP production rate (g/h)}=M_c/(V_{total}/Q_{total}) *60 \qquad \text{(Eq. S1)}$$

$$BaSO_4 \text{ NP yield (\%)}=M_c/M_i*100\% \qquad \text{(Eq. S2)}$$

Where V_{total}=20 mL and Q_{total}=281.4 mL/min. M_c is the quality (g) of the dried $BaSO_4$ NPs collected in the bottle. M_i is the theoretical generation quality (g) of $BaSO_4$ NPs when $BaCl_2$ and Na_2SO_4 react completely. Table S3 shows the result of production rates and yields at different reactant concentrations.

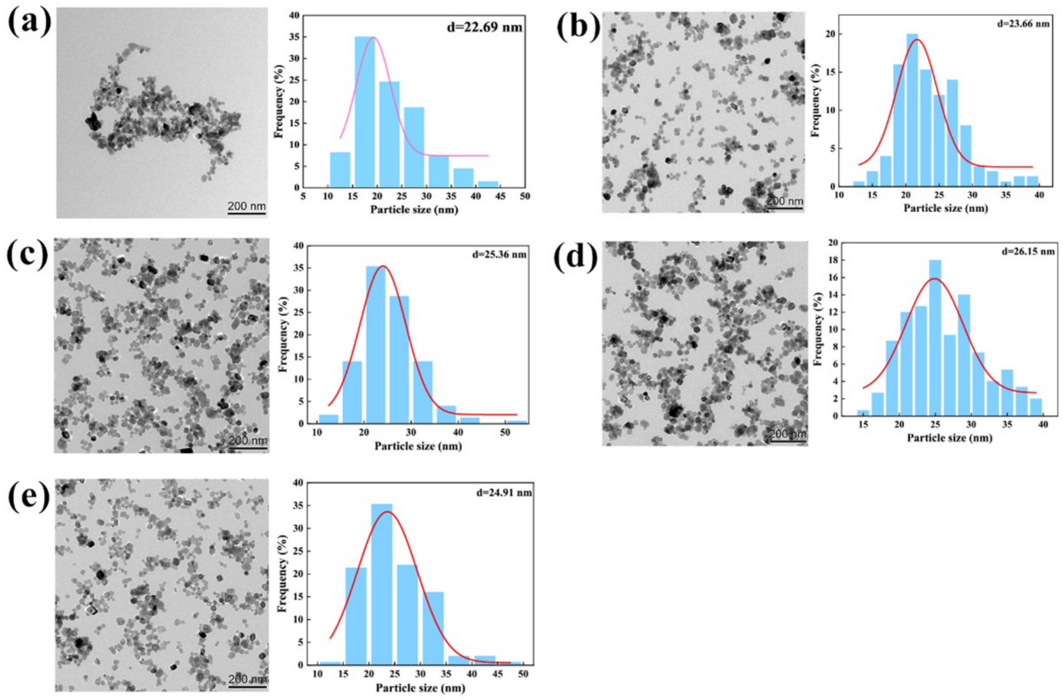

Fig. S5 Morphologies and size distributions of NPs synthesized in 4X OFM with different concentrations (a) 0.1 moL/L; (b) 0.15 moL/L; (c) 0.2 moL/L; (d) 0.25 moL/L; (e) 0.3 moL/L. The concentrations of $BaCl_2$ and Na_2SO_4 are the same.

Table S1 Comparison between OFM reactor and other reactors in terms of throughput.

Reactor		Flow patterns	Throughput (mL/min)
Disordered flow microreactors	OFM	Chaotic convection	160[3]
	OFM with loop	Chaotic convection	~19.6[4] (feed)
	Corning advanced-flow microreactor	Chaotic convection	1-9[5]
Ordered flow microreactors	Acoustophoresis microreactor	Laminar flow	0.4-1.2[6]
	Ultrasonic microreactor	Laminar flow	2[7]
		Segment flow	3.2-4[7]
	Ultrasonic microreactor	Laminar flow	0.2[8]
	Capillary microreactor	Segment flow	2.75-6.5[9]
	Segment flow microreactor	Segment flow	2.33-10[10]
	T-junction micromixers	Segment flow	0.8[11]
	Homemade capillary microreactor	Segment flow	0.5[12]

Table S2 Original data of nanoparticle size distribution obtained from TEM images in Fig.s 6, 8, and 10.

Number	Particle size (nm)							
	Fig. 6(a)	Fig. 6(b)	Fig. 6(c)	Fig. 8(a)	Fig. 8(b)	Fig. 8(c)	Fig. 10(a)	Fig. 10(b)
1	69.985	42.266	30.479	20.927	11.725	12.289	11.33	10.943
2	71.703	43.434	31.902	24.14	12.653	12.717	12.637	11.021
3	84.929	46.404	34.543	24.772	12.883	13.171	12.813	12.815
4	86.968	56.951	36.715	25.693	13.332	13.171	13.086	13.319
5	101.9	58.093	43.223	26.062	13.387	13.286	13.968	13.464
6	102.69	62.052	43.698	26.062	13.55	13.646	13.982	13.941
7	113.009	63.122	47.064	27.023	14.35	13.646	13.982	14.005
8	122.946	66.696	47.761	27.672	15.186	13.646	14.158	14.057
9	125.729	67.345	51.404	27.934	15.215	13.869	14.471	14.787
10	126.428	68.225	55.274	28.064	15.33	14.07	14.595	14.804
11	128.266	71.897	60.618	28.37	15.718	14.286	14.671	14.891
12	129.539	71.497	66.092	28.37	15.941	14.708	14.94	15.366
13	130.796	73.153	66.419	29.457	16.014	14.725	15.06	16.061
14	133.925	76.227	69.205	30.174	16.591	15.017	15.208	16.095
15	137.985	78.986	70.167	30.31	16.811	15.017	15.722	16.197
16	139.903	78.285	72.354	30.37	16.811	15.135	15.722	16.321
17	142.43	83.203	72.71	30.668	17.217	15.6	15.742	16.327
18	147.15	84.162	72.71	31.256	17.353	15.666	15.907	16.334
19	150.475	86.834	73.262	31.719	17.696	15.731	15.961	16.374
20	154.62	86.925	74.158	31.891	17.721	15.731	16.251	16.546
21	157.173	90.419	75.208	32.076	17.878	15.86	16.3	16.561
22	158.085	90.569	77.772	32.499	17.919	15.972	16.312	16.583
23	170.659	90.659	79.349	32.569	18.017	16.614	16.357	16.657
24	174.602	90.262	81.378	32.611	18.017	16.614	16.357	16.667
25	175.977	91.758	81.606	33.288	18.404	16.66	16.403	16.794
26	179.577	92.9	82.336	33.736	18.908	16.66	16.792	16.892
27	183.866	96.877	84.184	33.763	18.97	16.736	17.202	17.102
28	187.637	98.321	85.353	33.763	18.97	16.858	17.213	17.192
29	191.928	98.196	90.426	33.884	19.063	16.978	17.288	17.267
30	193.949	99.337	91.684	34.138	19.094	16.978	17.342	17.686
31	198.129	101.459	91.999	34.165	19.178	17.261	17.658	17.866
32	198.561	102.701	93.225	34.47	19.224	17.438	17.704	17.922
33	205.588	104.32	95.099	34.942	19.542	17.742	17.728	17.98
34	206.096	104.439	95.24	35.085	19.55	17.857	17.784	18.016
35	208.916	108.913	95.845	35.189	19.58	18.028	17.91	18.034
36	209.016	109.957	97.161	35.318	19.58	18.07	18.065	18.06
37	212.152	109.537	100.246	35.728	19.632	18.07	18.321	18.203
38	214.711	110.432	100.308	35.767	19.729	18.183	18.362	18.256
39	215.19	111.545	101.199	35.767	19.878	18.211	18.453	18.256
40	215.643	112.875	102.103	35.856	20.062	18.309	18.614	18.262

41	217.035	117.006	104.737	35.919	20.062	18.695	18.873	18.407
42	228.732	118.708	110.865	36.335	20.12	18.749	18.873	18.53
43	231.079	119.183	111.783	36.522	20.171	18.858	18.873	18.568
44	247.797	119.2	111.922	36.82	20.403	18.871	18.951	18.613
45	256.472	128.572	112.336	36.87	20.41	19.019	19.049	18.669
46	257.767	129.025	117.65	37.128	20.432	19.019	19.286	18.769
47	264.819	129.499	117.956	37.324	20.639	19.166	19.325	18.837
48	269.368	130.85	119.262	37.845	20.745	19.219	19.36	18.858
49	296.755	132.979	123.697	37.845	20.858	19.233	19.501	19.065
50	305.744	133.314	125.289	38.001	20.858	19.233	19.545	19.155
51	318.713	137.832	128.774	38.465	20.922	19.299	19.552	19.37
52	342.918	139.777	131.503	38.982	21.048	19.339	19.641	19.424
53	343.822	139.6	133.063	39.122	21.083	19.431	19.647	19.453
54	362.859	139.733	135.835	39.134	21.11	19.496	19.723	19.47
55	393.054	146.519	135.835	39.134	21.387	19.653	20.041	19.527
56	394.77	147.917	135.979	39.504	21.442	19.704	20.44	19.573
57	397.836	147.073	148.1	39.539	21.585	19.898	20.495	19.573
58	413.842	150.439	150.362	39.711	21.585	19.923	20.51	19.906
59	415.223	150.69	152.034	39.768	21.68	20.013	20.826	20.007
60	433.726	162.165	158.079	39.814	21.687	20.051	21.084	20.023
61	438.634	171.23	160.534	40.088	21.707	20.127	21.087	20.228
62	443.005	173.037	162.204	40.088	21.822	20.203	21.19	20.322
63	452.235	177.084	164.598	40.269	21.822	20.203	21.435	20.449
64	456.694	177.173	167.168	40.539	21.849	20.203	21.443	21.146
65	495.055	177.967	167.716	40.841	21.95	20.203	21.608	21.429
66	518.957	181.917	171.339	41.031	22.063	20.203	21.7	21.51
67	621.107	183.649	172.09	41.075	22.229	20.228	21.859	21.704
68	630.507	185.897	176.777	41.13	22.249	20.454	22.077	21.789
69	768.771	187.673	181.978	41.296	22.249	20.454	22.187	21.994
70	775.483	190.903	184.519	41.296	22.249	20.466	22.198	22.038
71	836.672	191.965	188.52	41.428	22.282	20.466	22.254	22.085
72		191.623	188.892	41.472	22.282	20.616	22.299	22.305
73		196.127	190.508	41.735	22.301	20.763	22.346	22.523
74		212.91	192.341	41.822	22.328	20.763	22.39	22.693
75		215.508	197.431	42.082	22.407	20.763	22.432	22.903
76		217.692	198.505	42.373	22.459	20.8	22.545	22.906
77		219.714	210.953	42.556	22.544	20.862	22.914	22.906
78		223.532	219.785	42.598	22.648	20.862	22.958	23.324
79		223.666	236.616	42.673	22.751	20.862	23.24	23.404
80		226.417	269.246	42.673	22.79	21.008	23.291	23.423
81		235.746	273.446	42.726	22.848	21.008	23.368	23.473
82		239.264	286.961	42.769	22.867	21.008	23.411	23.515
83		240.246	306.496	42.886	23.002	21.093	23.516	23.624
84		243.813	309.552	42.886	23.059	21.153	23.532	23.624
85		250.539	333.234	42.981	23.078	21.201	23.535	23.862
86		254.143	373.53	43.119	23.174	21.201	23.661	23.867
87		259.884	396.752	43.602	23.199	21.237	23.701	23.903

88		260.832	404.369	43.935	23.382	21.237	23.974	24.332
89		261.581	429.751	43.955	23.513	21.429	24.74	24.339
90		262.761	578.293	43.986	23.532	21.44	24.74	24.46
91		275.365		44.028	23.613	21.771	24.76	24.514
92		279.868		44.059	23.632	21.806	24.83	25.255
93		282.507		44.11	23.632	22.189	24.97	25.299
94		300.889		44.11	23.762	22.315	25.188	25.441
95		305.012		44.511	23.854	22.418	25.392	25.593
96		314.487		44.552	23.854	22.418	25.47	25.785
97		317.524		44.725	23.959	22.554	25.657	25.978
98		317.709		44.725	24.002	22.588	25.819	26.295
99		334.652		45.04	24.075	22.588	25.879	26.41
100		336.716		45.131	24.148	22.7	25.924	26.579
101		338.115		45.612	24.172	22.7	25.944	26.608
102		345.863		45.901	24.203	22.79	26.011	26.918
103		346.254		46.197	24.39	23.035	26.345	26.922
104		351.984		46.197	24.486	23.035	26.461	27.129
105		353.995		46.785	24.51	23.134	26.515	27.388
106		380.567		46.94	24.54	23.234	26.592	27.723
107		416.46		47.076	24.54	23.255	26.875	28.072
108		429.903		47.134	24.719	23.255	26.905	28.15
109		467.58		48.137	24.719	23.266	26.995	28.43
110		504.603		48.702	24.725	23.398	27.199	28.627
111		507.436		48.767	24.985	23.43	27.275	28.688
112		546.434		49.49	25.009	23.582	27.461	28.838
113				49.811	25.283	23.658	27.601	29.607
114				49.811	25.388	23.744	27.87	29.652
115				49.811	25.543	23.744	27.876	29.743
116				50.093	25.566	23.787	27.905	29.994
117				50.644	25.584	24	28.07	30.222
118				50.869	25.618	24	28.072	30.338
119				51.083	25.71	24.254	28.078	30.952
120				51.721	25.71	24.328	28.116	31.46
121				52.063	25.841	24.328	28.118	31.931
122				52.307	25.858	24.328	28.415	32.511
123				53.059	25.886	24.432	28.668	33.036
124				53.512	25.909	24.432	29.098	33.539
125				53.512	25.954	24.578	29.166	33.848
126				54.129	26.028	24.578	29.175	34.074
127				54.129	26.061	24.599	29.209	35.283
128				54.398	26.219	24.754	29.79	35.318
129				55.981	26.269	24.846	30.336	36.67
130				56.128	26.342	24.877	30.411	36.681
131				56.933	26.608	24.949	31.054	37.057
132				57.188	26.647	25.041	31.202	37.994
133				57.829	26.773	25.081	31.661	40.287
134				60.597	26.779	25.122	32.109	42.499

135				63.782	27.003	25.163	32.113	
136				64.864	27.391	25.274	32.434	
137				65.291	27.407	25.274	32.787	
138				66.178	27.407	25.274	32.806	
139				66.178	27.536	25.274	33.267	
140				66.178	27.637	25.334	33.557	
141				67.039	27.637	25.435	35.239	
142				69.65	27.664	25.525	35.793	
143				69.663	27.785	25.575	36.481	
144				71.105	28.001	25.764	36.8	
145				72.826	28.043	25.803	39.99	
146				75.436	28.361	25.892	40.99	
147				76.556	28.454	26	43.167	
148				78.945	28.619	26.069	46.704	
149				79.771	28.619	26.283	53.854	
150					28.619	26.745		
151					28.639	26.84		
152					28.665	26.84		
153					28.726	26.954		
154					29.127	27.002		
155					29.188	27.152		
156					29.393	27.479		
157					29.578	27.784		
158					29.652	27.793		
159					29.672	28.14		
160					29.672	28.302		
161					29.766	28.329		
162					29.766	28.446		
163					29.825	28.464		
164					29.85	28.571		
165					29.855	28.571		
166					30.299	28.58		
167					30.396	28.687		
168					30.526	28.794		
169					30.545	28.794		
170					30.66	28.9		
171					30.761	28.926		
172					30.818	29.146		
173					30.818	29.155		
174					30.899	29.181		
175					30.97	29.321		
176					31.159	29.321		
177					31.366	29.77		
178					31.604	30.009		
179					31.923	30.372		
180					32.093	30.456		
181					32.193	30.673		

182					32.416	30.673		
183					32.489	30.814		
184					32.633	31.012		
185					32.741	31.119		
186					32.969	31.119		
187					33.009	31.241		
188					33.08	31.623		
189					33.504	31.896		
190					33.735	31.984		
191					33.84	32.103		
192					34.129	32.103		
193					34.979	32.467		
194					35.13	32.576		
195					35.168	32.639		
196					35.176	33.579		
197					35.645	33.882		
198					36.168	33.882		
199					36.681	34.024		
200					36.685	34.077		
201					37.079	34.144		
202					37.087	34.293		
203					38.391	34.883		
204					38.578	35.664		
205					38.578	35.949		
206					38.589	36.056		
207					39.092	36.147		
208					39.28	36.422		
209					39.447	36.957		
210					39.459	37.253		
211					40.013	37.253		
212					40.502	37.253		
213					41.085	37.253		
214					41.257	37.253		
215					41.367	37.253		
216					42.377	37.253		
217					44.196	37.253		
218					44.829	37.253		
219					45.904	37.253		
220					45.917	37.266		
221					47.204	38.598		
222					48.793	38.717		
223					51.593	39.227		
224						41.607		
225						42.917		
226						44.544		
227						45.378		
228						45.815		

229						52.314		

Table S3 The NP production rates and yields in 4X OFM with different concentrations (total throughput, 281.4mL/min; *Re*=3178)

$BaCl_2$ (moL/L)	Na_2SO_4 (moL/L)	Theoretical NP quality M_i (g)	Actual NP quality M_C (g)	Yield	Production rate (g/h)
0.1	0.1	0.233	0.2125	91.2%	179.4
0.15	0.15	0.3495	0.32129	91.93%	271.23
0.2	0.2	0.466	0.4347	93.28%	366.97
0.25	0.25	0.5825	0.52736	90.53%	445.20
0.3	0.3	0.699	0.63774	91.24%	538.38

Screenshots Of Movie Clips

The images below are screenshots of Movie clips.

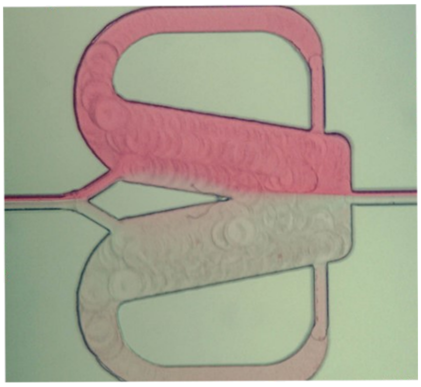

Video S1 Movie of flow pattern in 1X OFM: $Q_R = Q_L = 0.5$ mL/min: stable laminar flow (it seems to be stationary)

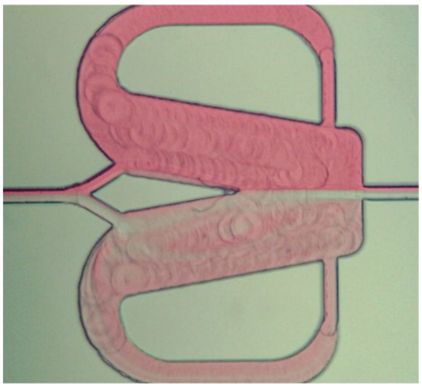

Video S2 Movie of flow pattern in 1X OFM: $Q_R = Q_L = 2$ mL/min: vortex flow and

feedback flow

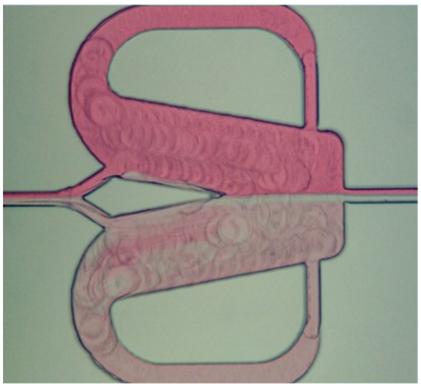

Video S3 Movie of flow pattern in 1X OFM: $Q_R = Q_L = 5$ mL/min: vortex flow and

feedback flow

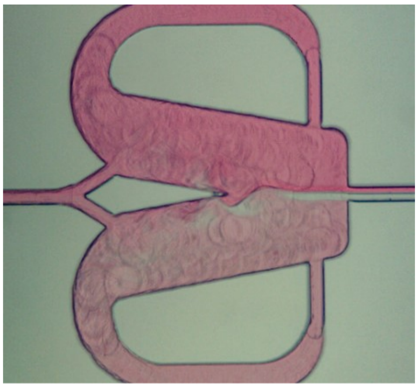

Video S4 Movie of flow pattern in 1X OFM: $Q_R = Q_L = 12.5$ mL/min: oscillating flow

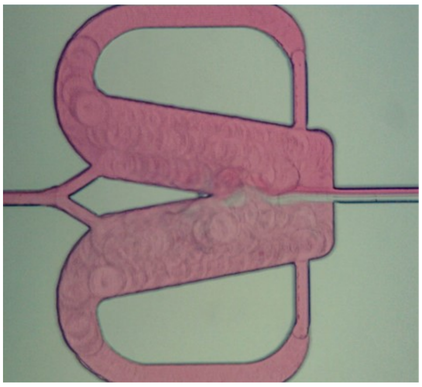

Video S5 Movie of flow pattern in 1X OFM: $Q_R = Q_L = 28.3$ mL/min: oscillating flow

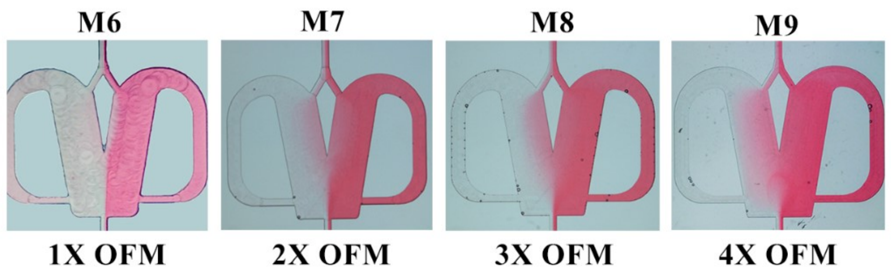

Video S6- Video S9 Movies of flow patterns in 1X OFM – 4X OFM when *Re* = 16.94:

laminar flow

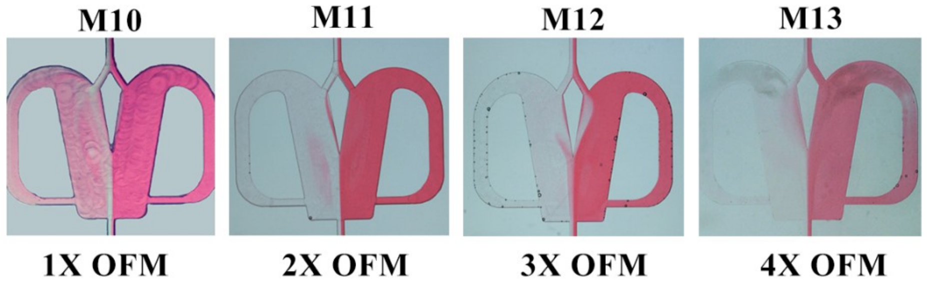

Video S10- Video S13 Movies of flow patterns in 1X OFM – 4X OFM when *Re* = 67.76:

vortex flow and feedback flow

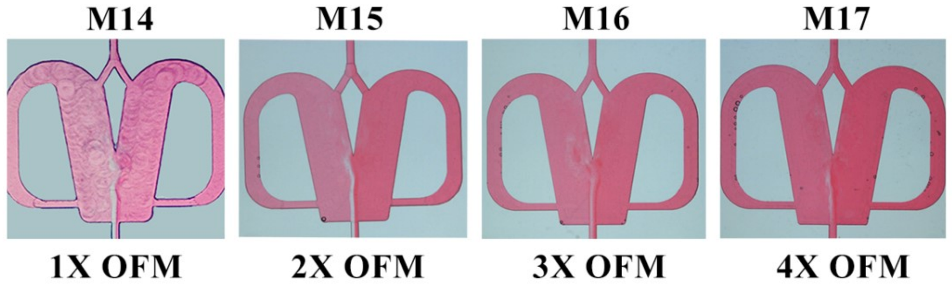

Video S14- Video S17 Movies of flow patterns in 1X OFM – 4X OFM when *Re* = 960.1:

oscillating flow

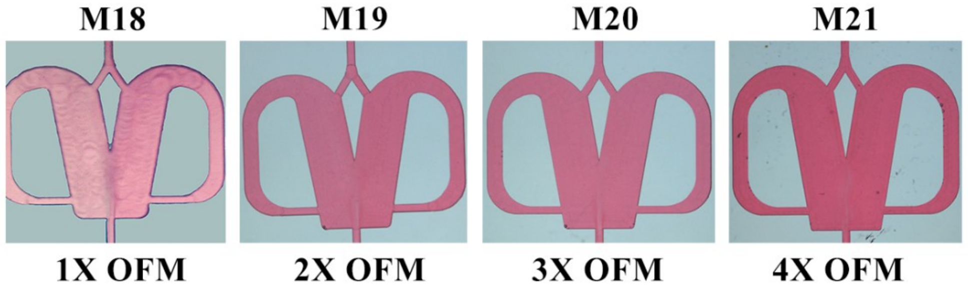

Video S18- Video S21 Movies of flow patterns in 1X OFM – 4X OFM when *Re* = 1920:

oscillating flow

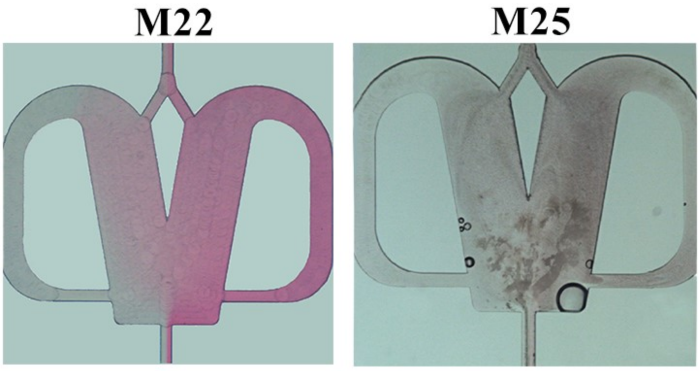

Video S22 and Video S25 Movies of flow patterns with and without NP synthesis in 2X OFM: $Q_R = Q_L = 0.07$ mL/min (laminar flow synthesis)

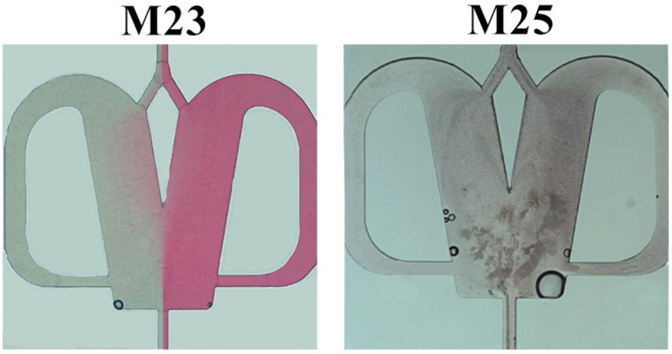

Video S23 and Video S26 Movies of flow patterns with and without NP synthesis in 2X OFM: $Q_R = Q_L = 0.7$ mL/min (laminar flow synthesis)

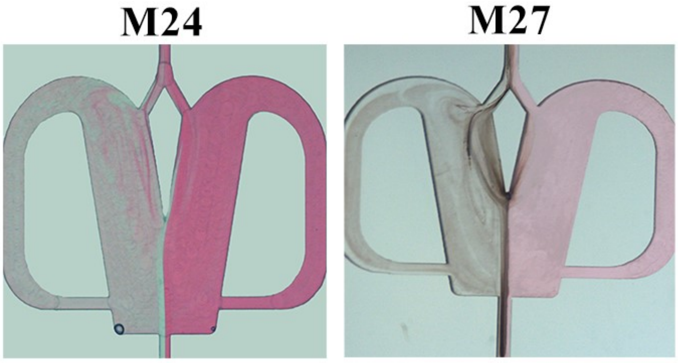

Video S24 and Video S27 Movies of flow patterns with and without NP synthesis in 2X OFM: $Q_R = Q_L = 7$ mL/min (vortex and feedback flow synthesis)

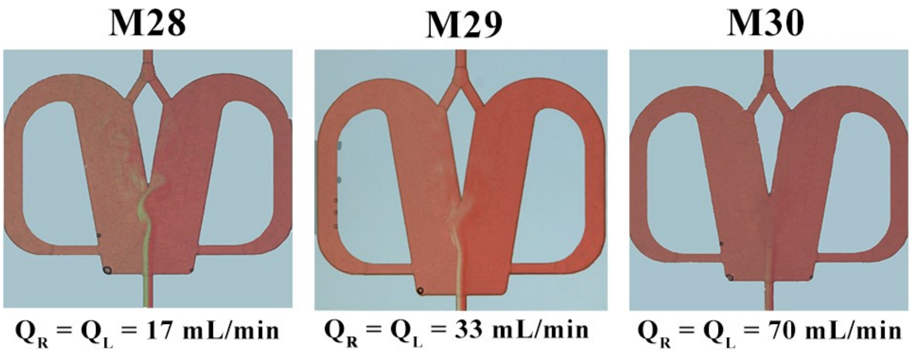

Video S28- Video S30 Movies of flow patterns without NP synthesis in 2X OFM: (a) $Q_R = Q_L$ = 17 mL/min; (b) $Q_R = Q_L$ = 33 mL/min; (c) $Q_R = Q_L$ = 70 mL/min (chaotic convection mode, i.e., intense oscillating flow)

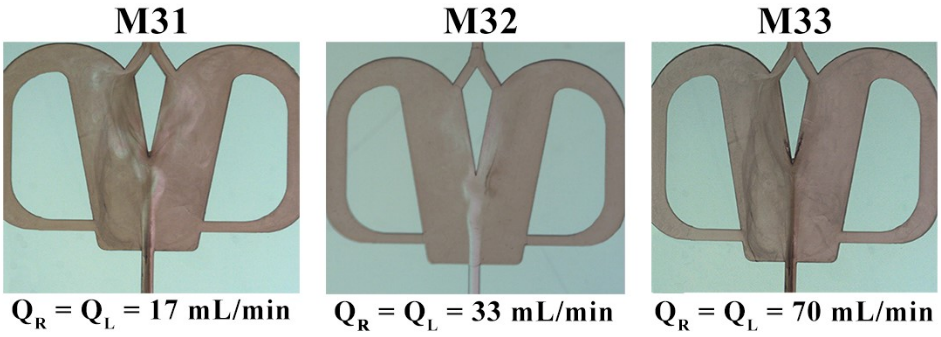

Video S31- Video S33 Movies of flow patterns with NP synthesis in 2X OFM: (a) $Q_R = Q_L$ = 17 mL/min; (b) $Q_R = Q_L$ = 33 mL/min; (c) $Q_R = Q_L$ = 70 mL/min (chaotic convection synthesis)

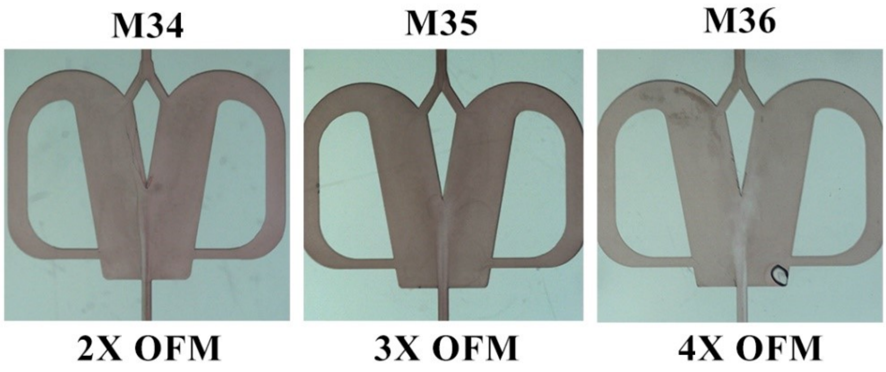

Video S34- Video S36 Movies of flow patterns with NP synthesis in 2X OFM – 4X OFM when *Re* = 1920 (chaotic convection synthesis)

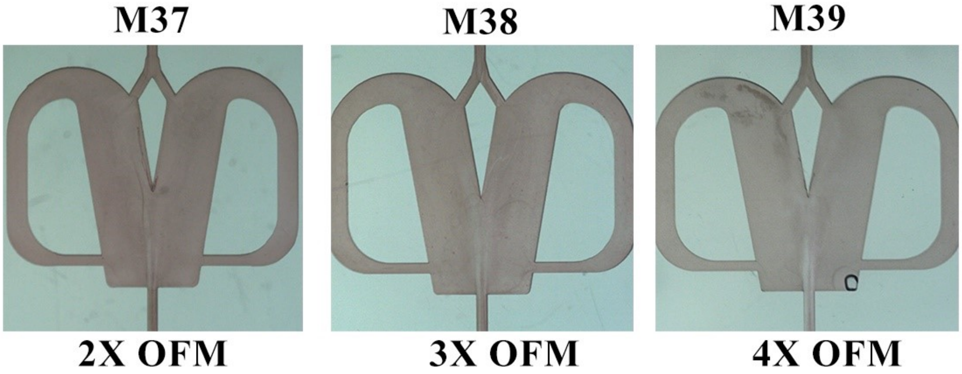

Video S37- Video S39 Movies of flow patterns with NP synthesis in 2X OFM – 4X OFM when *Re* = 3178 (chaotic convection synthesis)

References:

1. M. Yang, L. Yang, J. Zheng, N. Hondow, R. A. Bourne, T. Bailey, G. Irons, E. Sutherland, D. Lavric and K. J. Wu, *Chemical Engineering Journal*, 2021, **412**, 128565.
2. M. Yang, L. Luo and G. Chen, 2020, **66**, e16950.
3. H. Yang, S. X. Wei, H. Chen, L. Chen, C.T. Au, T. L. Xie and S. F. Yin, 2022, **68**, e17810.
4. C. Xu and Y. F. Chu, 2015, **61**, 1054-1063.
5. T. L. Xie, M. Chen, C. Xu and J. Chen, *Chemical Engineering Journal*, 2019, **356**, 382-392.
6. Y. F. Su, H. Kim, S. Kovenklioglu and W. Y. Lee, *Journal of Solid State Chemistry*, 2007, **180**, 2625-2629.
7. N. Sen, V. Koli, K. K. Singh, L. Panicker, R. Sirsam, S. Mukhopadhyay and K. T. Shenoy, *Chemical Engineering and Processing - Process Intensification*, 2018, **125**, 197-206.
8. A. V. Pandit and V. V. Ranade, *Industrial & Engineering Chemistry Research*, 2020, **59**, 3996-4006.
9. D. Jeevarathinam, A. K. Gupta, B. Pitchumani and R. Mohan, *Chemical Engineering Journal*, 2011, **173**, 607-611.
10. Z. Dong, D. Fernandez Rivas and S. Kuhn, *Lab on a Chip*, 2019, **19**, 316-327.
11. C. Delacour, C. Lutz and S. Kuhn, *Ultrasonics Sonochemistry*, 2019, **55**, 67-74.
12. F. Castro, S. Kuhn, K. Jensen, A. Ferreira, F. Rocha, A. Vicente and J. A. Teixeira, *Chemical Engineering Journal*, 2013, **215-216**, 979-987.
